# Study of trunk asymmetry in normal children and adolescents

**DOI:** 10.1186/1748-7161-1-19

**Published:** 2006-11-30

**Authors:** Theodoros B Grivas, Elias S Vasiliadis, Georgios Koufopoulos, Dimitrios Segos, Georgios Triantafyllopoulos, Vasilios Mouzakis

**Affiliations:** 1 Orthopaedic Department, "Thriasio" General Hospital, G. Gennimata Av. 19600, Magoula, Attica, Greece

## Abstract

The scoliometer readings in both standing and sitting position of 2071 children and adolescents (1099 boys and 972 girls) aged from 5 to 18 years old were studied. The angle of trunk rotation (ATR) was measured, in order to quantify the existing trunk asymmetry. Children and adolescents were divided in two groups according to the severity of trunk asymmetry. In the first group asymmetry was 1 to 6 degrees and in the second group was 7 or more degrees. Radiographic and leg length inequality evaluation were also performed in a number of children. The mean frequency of symmetric (ATR = 0 degrees) boys and girls was 67.06% and 65.01% for the standing screening position and 76.5% and 75.1% for the sitting position, respectively. The mean difference of frequency of asymmetry (ATR > 0 degrees) at standing minus sitting forward bending position for boys and girls was 10.22% and 9.37%, respectively. The mean frequency of asymmetry of 7 or more degrees was 3.23% for boys and 3.92% for girls at the standing forward bending position and 1.62% and 2.21% at the sitting, respectively. Girls are found to express higher frequency of asymmetry than boys. Right trunk asymmetry was more common than left. The sitting position is the preferred screening position for examining the rib or loin hump during school screening as it demonstrates the best correlation with the spinal deformity exposing the real trunk asymmetry.

## Background

The strongest indicator for referral and further orthopaedic assessment of the general children and adolescent population for scoliosis during school-screening is the amount of asymmetry of the trunk shape, which is the existence of a hump at the thoracic, thoracolumbar or lumbar area [1,2].

It is axiomatic, that any screening procedure for abnormality is based on knowledge of normality [3]. In this cross sectional study, the authors studied the trunk asymmetry in a sample of "normal" Mediterranean school children and adolescents. The distribution of trunk asymmetry by age was determined using a scoliometer. The 95% confidence limits of the method in use are set. The preferred position of examination for trunk asymmetry, that is the standing versus the sitting forward bending position, is determined. The incidence of small curves based on scoliometer readings is also reported.

## Materials and methods

The scoliometer measurements in 2071 children and adolescents with a range of age from 5.5 to 18 years were studied. There were 1099 boys (53.1%) and 972 girls (46.9%). The children and adolescents were examined at school, using a particular protocol, which involved record of demographics, somatometric characteristics, assessment of handedness and assessment of back shape morphology using the Pruijs scoliometer [4] (Orthomet-Surgeyplant B.V. Postbus 483, 5140 AL Waalwijl, Netherlands).

The bending test was performed in both standing and sitting forward bending position. In the standing forward bending position, the student was asked to bend forward looking down, keeping the feet approximately 15 cm apart, knees braced back, shoulders loose and hands positioned in front of knees or shins with elbows straight and palms opposed. Any leg length inequality was not corrected. The scoliometer was used at three areas of interest: at thoracic (T4–T8), thoraco-lumbar (T12-L1) and at the lumbar area (L3–L5). In the sitting forward bending position, the student was seated on a chair (40 cm high) and was asked to bend forwards and place the head between the knees with the shoulders loose, elbows straight and hands positioned between knees. The scoliometer measurements were obtained successively at the same three areas of interest as in the standing forward bending position.

Scoliometer measurement equal to 0° was defined as symmetry at the particular level of the trunk. Any other scoliometer value was defined as asymmetry.

**Figure 1:**
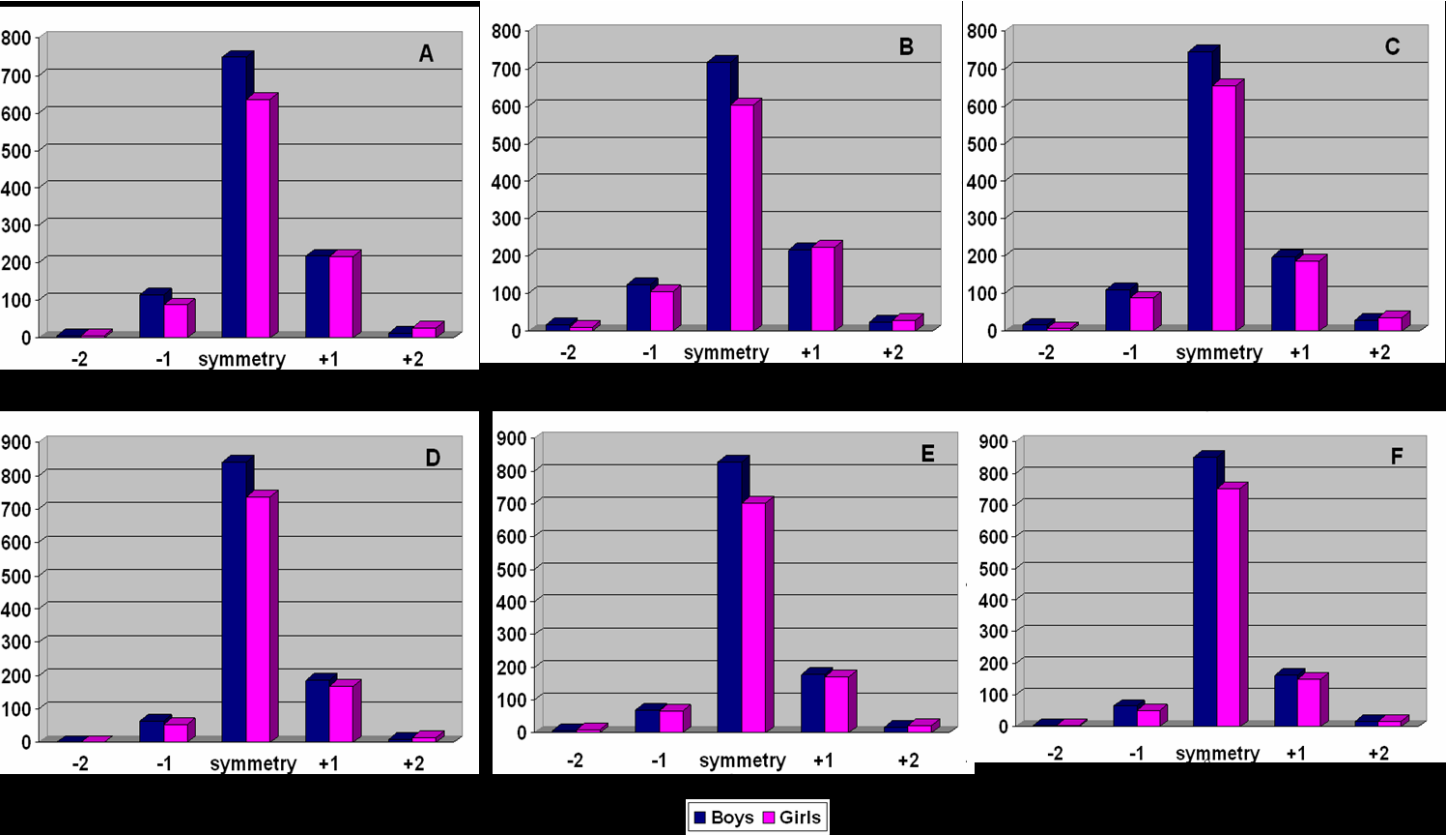
Column charts showing trunk asymmetry of boys and girls at the three examined regions. A. Scoliometer readings in the *thoracic *region at *standing forward bending *position. B. Scoliometer readings in the *thoracolumbar *region at *standing forward bending *position. C. Scoliometer readings in the *lumbar *region at *standing forward bending *position. D. Scoliometer readings in the *thoracic *region at *sitting forward bending *position. E. Scoliometer readings in the *thoracolumbar *region at *sitting forward bending *position. F. Scoliometer readings in the *lumbar *region at *sitting forward bending *position. +1: Scoliometer readings from 1° to 6° to the right. -1: Scoliometer readings from 1° to 6° to the left. +2: Scoliometer readings ≥7° to the right. -2: Scoliometer readings ≥7° to the left.

According to the severity of the trunk asymmetry, children and adolescents were divided into two groups. In the first group, the scoliometer readings were less than 7° and in the second group the scoliometer readings were 7° or more. Right asymmetry was deemed plus (+) and left asymmetry was deemed minus (-), Figure 1.

All children and adolescents of the second group were referred at hospital for further clinical and radiological examination. In children and adolescents with scoliometer measurements of 5 or 6 degrees and strong clinical suspicion of scoliosis, radiological assessment was also done.

Leg length inequality (LLI) measurements were obtained regularly in referred patients at the Scoliosis Clinic and not as routine during school screening. With the patient in the supine position the distance between the anterior superior iliac spine and the tip of the ipsilateral medial malleolus was measured. The difference between left and right represents the LLI.

**Figure 2:**
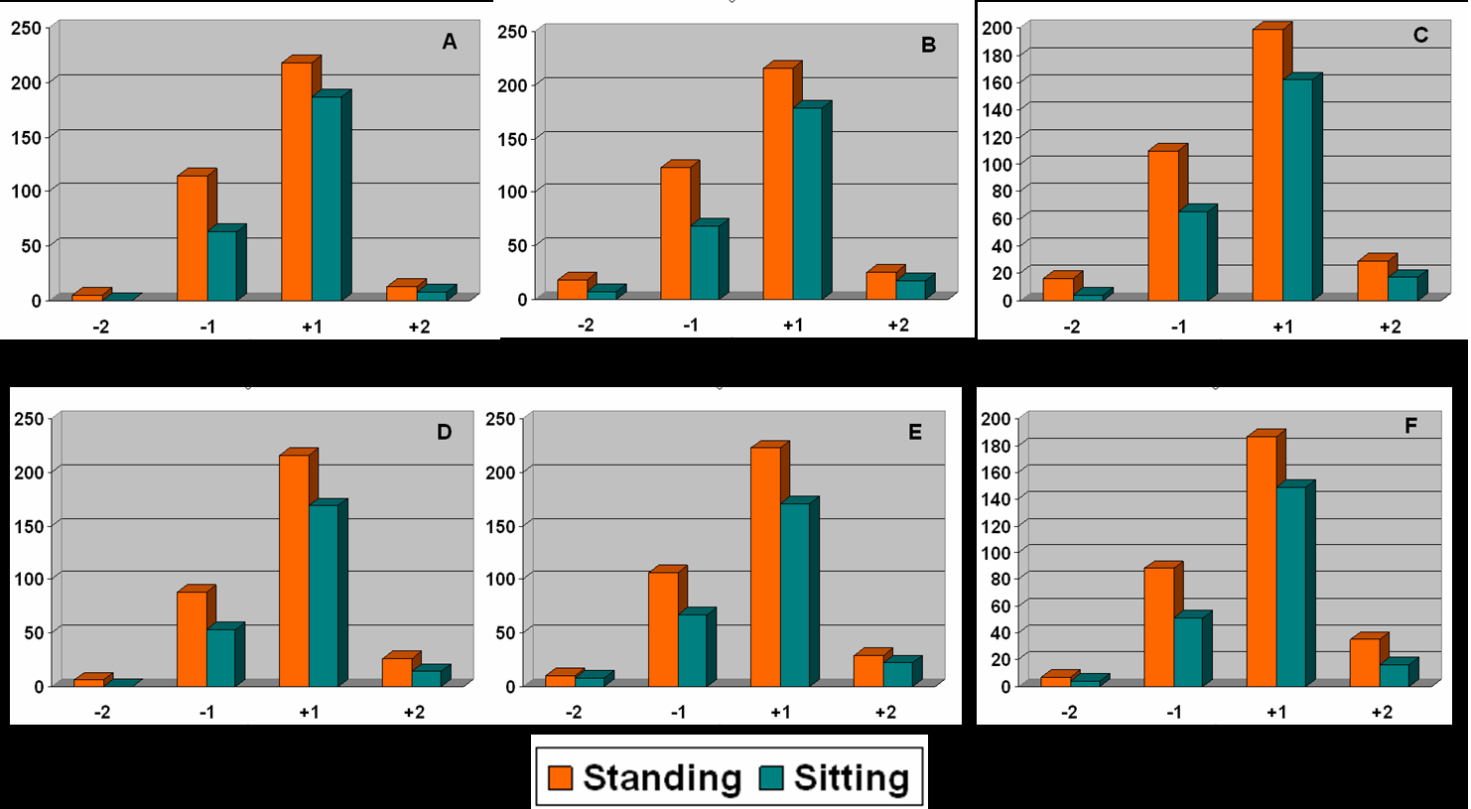
Column charts showing the difference in scoliometer readings between standing and sitting forward bending position at the three examined regions. A. Scoliometer reading differences in boys at the *thoracic *region. B. Scoliometer reading differences in boys at the *thoracolumbar *region. C. Scoliometer reading differences in boys in the *lumbar *region. D. Scoliometer reading differences in girls at the *thoracic *region. E. Scoliometer reading differences in girls at the *thoracolumbar *region. F. Scoliometer reading differences in girls at the lumbar region. +1: Scoliometer readings from 1° to 6° to the right. -1: Scoliometer readings from 1° to 6° to the left. +2: Scoliometer readings ≥7° to the right. -2: Scoliometer readings ≥7° to the left.

Some children and adolescents showed different scoliometer readings (Angle of Trunk Rotation, ATR) between standing and sitting screening position, Figure 2. The difference of the standing minus sitting ATR was defined as correction  of the ATR (CATR) for LLI.

Among the referred children and adolescents, 50 had LLI. In this sample the correlation between the CATR and LLI was studied. The Pearson's correlation coefficient between CATR and LLI was calculated.

Scoliometer readings on thoracic, thoracolumbar and lumbar region in both standing and sitting position of 10 randomly selected children aged within the range of study population were used. Asymmetry was measured twice by the same observer (TBG) on each child and intra-observer error was calculated within 95% confidence limits using the formula: ***error = SDx2/√2***, where SD is standard deviation. The inter-observer error was calculated using the readings of the first observer (TBG) and those of a second observer (ESV), using the same formula.

For the statistical analysis, the SPSS-v.11 statistical package was used. Statistical techniques included frequencies, descriptives, Pearson's Correlation Coefficient, Mann-Whitney and Wilcoxon tests, cross-tabulation and scatter plots.

## Results

Figure 1 demonstrates the trunk asymmetry assessed at both standing and sitting forward bending position at the three examined regions for boys and girls.

Figure 2 shows the difference in scoliometer readings between standing and sitting forward bending position at the three examined regions for boys and girls.

The range of leg length inequality among all the referred children was 0.5 to 3.2 cm.

The Pearson's correlation coefficient between the CATR and the LLI in the Thoracic, Thoracolumbar and Lumbar region was 0.654, 0.670, 0.729 respectively as shown in Table 1. There was a strong correlation between CATR and LLI, suggesting that the leg length inequality may force the trunk to rotate, so the body will maintain its balance.

**Table 1:** Pearson's correlation coefficient (r) between LLI and CATR in the three examined regions: the leg length inequality may force the trunk to rotate, so the body will maintain its balance.

	**Thoracic CATR**	**Thoracolumbar CATR**	**Lumbar CATR**
**LLI**	r = 0.654	r = 0.670	r = 0.729

Reliability study revealed that intraobserver error was 1.82°, 3.24° and 4.04° for standing scoliometer readings and interobserver error was 2.63°, 3.33° and 4.29° for standing scoliometer readings at the thoracic, thoracolumbar and lumbar region respectively. In the sitting position intraobserver error was 2.15°, 2.55° and 4.09° and interobserver error was 3.73°, 2.13° and 4.10° at the thoracic, thoracolumbar and lumbar region respectively.

Analyzing our reliability study for Pruijs's scoliometer measurements, Table 2, we observe that maximal variability is shown at the lumbar and minimal at the thoracic region. Scoliometer measurements are reported to have a variability of ± 2° to ± 4° [5].

**Table 2:** Reliability study for Pruijs's scoliometer readings.

**Scoliometer readings**	**Intra-observer error**	**Inter-observer error**
*standing forward bending test*		
Thoracic (T4-8)	1.82°	2.63°
Thoracolumbar (T11-L1)	3.24°	3.33°
Lumbar (L3-5)	4.04°	4.29°
*sitting forward bending test*		
Thoracic (T4-8)	2.15°	3.73°
Thoracolumbar (T11-L1)	2.55°	2.13°
Lumbar (L3-5)	4.09°	4.10°

The frequency of trunk asymmetry in boys after standing and sitting screening position is shown in Tables 3 and 4, respectively. The respective data of the examined girls are shown in Tables 5 and 6. The difference of frequency of the asymmetry in standing minus sitting position for the thoracic, thoracolumbar and lumbar region is shown Table 7.

**Table 3:** Frequency of asymmetry in *boys*. Scoliometer readings are at *standing *forward bending position.

	**Symmetry (%)**	**Asymmetry (%)**					
		**Total**	**-2**	**-1**	**+ 1**	**+2**	**(-2) + (+2)**

**Thoracic**	68.15	31.85	0.46	10.30	19.80	1.20	1.66
**Thoraco-lumbar**	65.27	34.73	1.67	11.14	19.59	2.32	3.99
**Lumbar**	67.78	32.22	1.48	10.02	18.10	2.59	4.07
**Mean**	67.06	32.94	1.20	10.48	19.16	2.03	3,23

In the first group (asymmetry from 1° to 6°) right asymmetry was deemed +1 and left asymmetry was deemed -1. In the second group (asymmetry ≥ 7°) right asymmetry was deemed +2 and left asymmetry was deemed -2

**Table 4:** Frequency of asymmetry in *boys*. Scoliometer readings are at *sitting *forward bending position.

	**Symmetry (%)**	**Asymmetry (%)**					
		**Total**	**-2**	**-1**	**+1**	**+2**	**(-2) + (+2)**

**Thoracicc**	76.50	23.50	0.00	05.75	16.99	0.74	0.74
**Thoraco-lumbar**	75.39	24.61	0.64	06.12	16.24	1.57	2.21
**Lumbar**	77.41	22.59	0.37	05.94	14.68	1.57	1.94
**Mean**	76.43	23.57	0.33	05.93	15.97	1.29	1.62

In the first group (asymmetry from 1° to 6°) right asymmetry was deemed +1 and left asymmetry was deemed -1. In the second group (asymmetry ≥ 7°) right asymmetry was deemed +2 and left asymmetry was deemed -2

**Table 5:** Frequency of asymmetry in *girls*. Scoliometer readings are at *standing *forward bending position.

	**Symmetry (%)**	**Asymmetry (%)**					
		**Tolal**	**-2**	**-1**	**+ 1**	**+2**	**(-2) + (+2)**

**Thoracic**	65.48	34.52	0.62	09.10	22.07	2.71	3.33
**Thoraco-lumbar**	62.17	37.83	1.04	10.86	22.88	3.03	4.07
**Lumbar**	67.39	32.61	0.73	09.09	19.12	3.65	4.38
**Mean**	65.01	34.99	0.79	09.68	21.35	3.13	3.92

In the first group (asymmetry from 1° to 6°) right asymmetry was deemed +1 and left asymmetry was deemed -1. In the second group (asymmetry ≥ 7°) right asymmetry was deemed +2 and left asymmetry was deemed -2.

**Table 6:** Frequency of asymmetry in *girls*. Scoliometer readings are at *sitting *forward bending position.

	**Symmetry (%)**	**Asymmetry (%)**					
		**Total**	**-2**	**-1**	**+ 1**	**+2**	**(-2) + (+2)**

**Thoracic**	75.75	24.25	0.00	05.43	17.34	1.46	1.46
**Thoraco-lumbar**	72.41	27.59	0.83	06.89	17.55	2.29	3.12
**Lumbar**	77.40	22.60	0.41	05.23	15.27	1.67	2.08
**Mean**	75.18	24.82	0.41	05.83	16.72	1.80	2.213

In the first group (asymmetry from 1° to 6°) right asymmetry was deemed +1 and left asymmetry was deemed -1. In the second group (asymmetry ≥ 7°) right asymmetry was deemed +2 and left asymmetry was deemed -2.

**Table 7:** Difference of % of asymmetry at standing minus sitting forward bending position for boys and girls.

	**Girls**	**Boys**	**Girls minus Boys**
**Thoracic**	10.27%	8.35%	1.92
**Thoracolumbar**	10.24%	10.17%	0.07
**Lumbar**	10.17%	9.63%	0.54
**Mean**	10.22	9.378	0.84

The frequency of the trunk asymmetries found in both boys and girls in the standing position is higher than the frequency found in the sitting position. Furthermore, the increase in the frequency of asymmetry is greater in girls than in boys as shown in table 7. This finding probably reflects the greater prevalence of scoliosis in girls [6-10].

To the current study, the mean difference of asymmetry frequency at standing minus sitting forward bending position for boys and girls in total was found to be 10.22% and 9.37%, respectively. The mean percentage of the second group of asymmetry at the three areas of interest, (thoracic, thoracolumbar and lumbar), for boys was 3.23% for the standing and 1.63% for the sitting foreword bending position and for girls 3.92% and 2.21%, respectively, Table 7. As it was evident from the radiological evaluation, these percentages reflect small curves of >10° Cobb angle.

All the 23 children and adolescents of group two showed a scoliotic curve with a Cobb angle > 10° in the standing posteroanterior radiograph of the entire vertebral column.

No spinal deformity exceeding 10° of Cobb angle was found after the radiological assessment of children with 5° and 6° of scoliometer readings and strong clinical suspicion of scoliosis.

## Discussion

### General

This study concerns a great number of healthy children and adolescents. This type of analysis in this report enables defining the normal limits of various parameters and gives values of trunk asymmetry. It respects the Bunnell's indications for a critical value of 7 degrees of the angle of trunk rotation in the scoliosis screening [2]. The results also argue for use of the sitting position in scoliosis screening. This study documents that the relative proportion of a spinal curvature that  is caused by a LLI can be detected by a simple method that easily can be  incorporated into screening protocols: ATR at forward bending is measured  with the subject in a seated position, and the values are compared with  those obtained while the subject is standing. The CATR was highly correlated  with LLI among patients screened.   This particular observation, as far as it could be searched in the available peer-reviewed literature, has not been documented among detailed biomechanical analysis [6,20-22].

This work has also implications not only for diagnosis and for understanding aetiology and prognosis, as it is inferred here, but also as an avenue to help make targeted decisions regarding referral of patients screened. The report also provides a starting point, potentially, for a new avenue for diagnostic screening, evaluation, and treatment based on simple analysis of the positional dynamics of torso deformity.

Trunk asymmetries (ATR > 0°) are found in normal children with no spinal curves, as it is shown in our study and in other reports [3,11]. Thus, scoliometer measurements of 1° – 6° are considered to be associated with nonscoliotic spines. However, children with 5° – 6° of scoliometer measurements are followed up clinically at the Scoliosis Clinic every 4–6 months.

It has been reported, that scoliotic curves of >10° Cobb angle were found in 2% of subjects 12–16 years of age [2,4,12-14]. This percentage is closer to that found in the current study of 7 degrees or more asymmetry group, in sitting forward bending position. In other scoliosis screening studies, the reported prevalence generally falls between 2.5 – 4% [15,16]. The asymmetries reflecting the percentage of small curves in the current study are similar to other reports from school screening for scoliosis performed by other scoliosis departments in Greece [8,17].

In a previous study of a relatively younger sample of children, the more frequently found asymmetries were those to the left, while the right-sided asymmetries were more frequently traced by age [18]. In the present sample of older children and adolescents, the frequency of asymmetries was always nearly twice larger to the right, for the population studied, except for the male age group of less than 6 years at the thoracic and lumbar area.

In the current study, asymmetries were detected in greater percentages at the standing than in the sitting foreword bending position. If asymmetry detected in the sitting position is considered as the true trunk asymmetry, the most frequent affected part of the spine (ATR > 0°) was the thoracolumbar part in either boys or girls (24.61% of boys, 27.59 % of girls). It has been reported that younger children show most frequently lumbar asymmetry [18]. In the current study, girls had a higher frequency of total trunk asymmetries than boys in both standing (34.99 % vs. 32.94%) and sitting (24.82% vs. 23.57%) screening position.

In the current study, there was a strong correlation of the CATR and the LLI, which means that the LLI may force the trunk to rotate so the body can maintain its balance. The dynamics of torso deformity have been measured and plotted in a variety of biomechanical approaches [19-22]. However, the level of the spine where this rotatory force mainly occurs and the biomechanical changes, which imposes on the spine, is an issue that will be answered by future studies.

Little information on LLI and scoliosis exists in the peer-review literature [23-28]. In healthy children a physiological shortening of one leg (1 – 2 cm) is associated with a contralateral hump on the back in forward flexion. It has been reported that shortening on the right is less common in boys than in girls [3]. Ingelmark and Lindstrom [29] reported that the right leg of adults is usually shorter than the left. Considering that most people preferentially use the left leg, its longer length could perhaps be ascribed to a growth acceleration induced by the greater working load imposed on it [29]. The left foot supports a significantly higher load than the right in right-handed subjects [30-32]. The typical asymmetric pelvis has also its left half set a little higher and further back than the right [29,30]. It has been reported that shortening of one lower limb is associated with contralateral hump on the back not only at L3 but also at T12 and T8 vertebrae [3]. This finding indicates that the standing forward bending position used as a routine in school screening, while satisfactory for clinical use, should be replaced by a standard sitting forward bending position when measurements are needed [3].

The differences of frequency of asymmetry in the examination in the two positions are probably expressing the existing small leg length inequalities, or in scoliotics the coupling phenomenon between "hump rotation" and forward flexion in lumbar lateral curves [33]. The examination of the back trunk shape, with the child placed in sitting forward bending position expresses real trunk asymmetry, which is revealed due to the leveling of the pelvis and elimination of any effect of leg length inequality on back shape. Thus this position is preferred over the standing forward bending position if true trunk asymmetry is the one to be assessed. The results of the current study warrant additional research to explore the hypothesis that the standard sitting forward bending position for examining the rib or loin hump during school screening is the preferred screening position and demonstrates the best correlation with the spinal deformity. However standing forward bending position alone, is inconsiderately appraising both trunk and leg length asymmetry when it is used as the main test in school screening program.

The pattern of scoliosis associated with LLI, (anisomelia), is usually described as being compensatory, non-structural and non-progressive. It has also been reported that anisomelia can produce structural changes in the adult spine with time and many of the patients are experiencing back pain [34,35].

Leg asymmetries in normal children are either equalized during growth, or with the contribution of other mechanisms, according to our hypothesis, facilitate the increase of trunk asymmetry and probably aetiological implications on the pathogenesis of scoliosis [36]. Ingelmark and Lindstrom [29] reported that the causation of scoliosis is very difficult to establish because it may involve a large number of different mechanisms acting singly or in combination. They suggest that probably the main factor among others is the usually longer right leg in children prior to puberty.

Spinal curvature is "expressed" into surface asymmetry via the rib cage, spinal muscles, viscera, fat, and skin in a manner that is unique to each patient and changes over time as the deformity progresses [19]. The findings of the current study and the above mentioned hypothesis suggests that trunk asymmetry as measured using the scoliometer could be the surface expression of the asymmetrical action of a "composite muscle trunk rotator", (part of Nottingham AIS theory) for pathogenesis of scoliosis [37].

Another statement that can be implied from our school-screening program is that asymmetries in the form of thoracic or lumbar hump are earlier traced in the thorax or in the loin, without any apparent deformity in the spine (central axis). This statement was verified in a number of radiographic examinations in our referrals, a study presented elsewhere [38]. This means that the deforming forces, which begin the asymmetry, do not start within the spine, as it is stated in other reports [39], but elsewhere.

## Authors' contributions

TBG was the principal investigator of the study, conducted the collection of data, performed the statistical analysis and involved in drafting the article and in the interpretation of data. ESV participated in the school screening program, helped in manuscript drafting and in the interpretation of data. GK, DS and GT participated in the school screening program and performed part of literature review. All the authors read and approved the final manuscript.

## References

[B1] Bunnell WP (1984). An objective criterion for scoliosis screening. J Bone Joint Surg.

[B2] Bunnell WP (1993). Outcome of Spinal Screening. Spine.

[B3] Burwell RG, James NJ, Johnson F, Webb JK, Wilson YG (1983). Standardized trunk asymmetry scores – a study of back contour in healthy children. J Bone Joint Surg.

[B4] Pruijs JEH, Keessen W, van der Meer R, van Wieringen JC, Hageman MAPE (1992). Schoolscreening for scoliosis. Methodological considerations. Part 1. Spine.

[B5] Mubarak SJ, Wyatt MP, Leach J (1984). Evaluation of the intra-examiner and inter-examiner reliability of the scoliometer in measuring trunk rotation. Proceedings of the 19th annual meeting of Scoliosis Research Society September 19–22, Orlando, Florida.

[B6] Chan A, Moller J, Vimbani G, Paterson D, Southwood R, Sutherland A (1986). The scoliosis screening in Australian adolescents. Med J Aust.

[B7] Palmisani M, Bettini N, Gargiulo G, Nardi S, Rizquallah Y, Cosco F, Savini R (1990). The epidemiology of idiopathic scoliosis in the city of Bologna: A three year review of positive cases. Chir Organi Mov.

[B8] Soucacos PN, Soucacos PK, Zacharis KC, Beris AE, Xenakis TA (1997). School screening for scoliosis. A prospective epidemiological study in northwestern and central Greece. J Bone and Joint Surg.

[B9] Weinstein SL (1994). Adolescent idiopathic scoliosis: Prevalence and natural history. The Pediatric Spine: Principles and Practice.

[B10] Nissinen MJ, Heliovaara MM, Seitsamo JT, Kononen MH, Hurmerinta KA, Poussa MK (2000). Development of trunk asymmetry in a cohort of children ages 11 to 22 years. Spine.

[B11] Vercauteren M, Van Beneden M, Verplaetse R, Croene P, Uyttendaele D, Verdonk R (1982). Trunk Asymmetries in a Belgian School Population. Spine.

[B12] Brooks HL, Zorab PA, Siegler D (1979). Current incidence of scoliosis in California. Scoliosis 1979 – Based on the proceedings of the sixth symposium on scoliosis held at the Cardiothoracic Institute Brompton Hospital London on 17th and 18th September.

[B13] Korovessis PG, Stamatakis MV (1996). Prediction of scoliotic Cobb angle with the use of the scoliometer. Spine.

[B14] Pruijs JEH (1998). Personal communication.

[B15] Samelis P, Chatziargyropoylos T, Schinas V, Theocharis N, Grivas TB, Grivas TB (2000). School Screening at Thriasio Pedio. School Screening in Greece.

[B16] Lonstein JE (1977). Screening for Spinal Deformities in Minnesota Schools. Clin Orthop.

[B17] Smyrnis PN, Valavanis J, Alexopoulos A, Siderakis G, Giannestras NJ (1979). School screening for scoliosis in Athens. J Bone Joint Surg.

[B18] Grivas TB, Nakos B, Kouvaras J, Kalamakis N, Polyzois D, Stokes IAF (1999). Study of the natural history of the back trunk shape by the use of scoliometer, in children aged 5–12 years. Research into spinal deformities 2.

[B19] Jaremko JL, Poncet P, Ronsky J, Harder J, Dansereau J, Labelle H, Zernicke RF (2002). Indices of torso asymmetry related to spinal deformity in scoliosis. Clin Biomech.

[B20] Ashton-Miller JA, Schultz AB (1988). Biomechanics of the human spine and trunk. Exercise & Sport Sciences Review.

[B21] Closkey RF, Schultz AB, Luchies CW (1992). A model for studies of the deformable rib cage. J Biomech.

[B22] Stokes IAF, Armstrong JG, Moreland MS (1988). Spinal deformity and back surface asymmetry in IS. J Orthop Res.

[B23] Fontanesi G, Giancecchi F, Rotini R (1987). Segmental shortening and equalization for leg length discrepancies in adults. Italian Journal of Orthopaedics & Traumatology.

[B24] Gibson PH, Papaioannou T, Kenwright J (1983). The influence on the spine of leg-length discrepancy after femoral fracture. J Bone Joint Surg.

[B25] Gurney B (2002). Leg length discrepancy. Gait & Posture.

[B26] Jones KB, Sponseller PD, Hobbs W, Pyeritz RE (2002). Leg length discrepancy and scoliosis and Marfan syndrome. J Ped Orthop.

[B27] Steen H, Terjesen T, Bjerkreim I (1997). Anisomelia. Clinical consequences and treatment. Tidsskrift for Den Norske Laegeforening.

[B28] Timgren J, Soinila S (2006). Reversible pelvic asymmetry: an overlooked syndrome manifesting as scoliosis, apparent leg-length difference, and neurologic symptoms. Journal of Manipulative & Physiological Therapeutics.

[B29] Ingelmark BE, Lindstrom J (1962). Asymmetries of the lower extremities and pelvis and their relations to lumbar scoliosis. Acta Morphol Scan.

[B30] Adams W (1865). Lectures on the Pathology and treatment of lateral and other forms of curvature of the Spine.

[B31] Marsk A (1958). Studies on weight-distribution upon the lower extremities in individuals working in a standing position: assessing the results of the measurements of load-pressure differences against the background of handedness and some clinical observations. Acta Orthop Scand.

[B32] Grivas TB, Needoff M, Kastrisios M, Burwell RG, Prince HG, Wallace WA (1992). Study of the stance phase of gait by the use of Musgrave plate in children aged 6–12 years. Acta Orthop Hellenica.

[B33] Upadhyay SS, Burwell RG, Nicholson UJL, Webb JK, Stokes IAF, Pekelsky JR, Moreland MS (1987). The integrated shape imaging system (ISIS) and the scoliometer for recording back shape in scoliosis. A reliability and comparative study revealing positional changes in back contour (hump dynamics). Surface Topography and spinal deformity IV.

[B34] Papaioannou T, Stokes I, Kenwright J (1982). Scoliosis associated with limb-length inequality. J Bone Joint Surg.

[B35] Scheller ML (1964). Uber den Einfluss der Beinverkurzung auf die Wirbelsaule. PhD Thesis.

[B36] Goldberg CJ, Moore DP, Fogarty EE, Dowling FE, Tanguy A, Peuchot B (2000). The relation between minor asymmetry and early Idiopathic Scoliosis. Research into Spinal Deformities 3.

[B37] Burwell RG, Cole AA, Cook TA, Grivas TB, Kiel AW, Moulton A, Thirlwall AS, Upadhyay SS, Webb JK, Wemyss-Holden SA, Whitwell DJ, Wojcik AS, Wythers DJ (1992). Pathogenesis of Idiopathic Scoliosis. The Nottingham Concept. Acta Orthop Belgica.

[B38] Grivas TB, Daggas S, Polyzois Bd, Samelis P (2002). The double rib contour sign (drcs) in lateral spinal radiographs. Aetiologic implications for scoliosis?. Stud Health Technol Inform.

[B39] Dickson RA, Lawton JO, Archer IA, Butt WP (1984). The pathogenesis of idiopathic scoliosis: biplanar spinal asymmetry. J Bone Joint Surg.

